# Screening and mechanistic study of natural compounds that enhance T cell anti-tumor effects post-heat treatment

**DOI:** 10.3389/fimmu.2025.1537398

**Published:** 2025-03-27

**Authors:** Zhaoyi Wang, Zhongqi Diao, Yiyan Zhang, Jiangying Liu, Yeshan Li, Zijin Sun, Huimin Zhen, Haojia Wang, Siyun Yang, Tieshan Wang, Lei Ni

**Affiliations:** ^1^ School of Traditional Chinese Medicine, Beijing University of Chinese Medicine, Beijing, China; ^2^ Department of Cancer Research Institute, Affiliated Cancer Hospital of Xinjiang Medical University, Urumqi, China; ^3^ School of Life Sciences, Beijing University of Chinese Medicine, Beijing, China; ^4^ School of Chinese Materia Medica, Beijing University of Chinese Medicine, Beijing, China; ^5^ Beijing Research Institute of Chinese Medicine, Beijing University of Chinese Medicine, Beijing, China

**Keywords:** adoptive cell therapy, Chinese medicine, temperature, proteomics, untargeted metabolomics, enhance immunity, RNA-Seq

## Abstract

**Introduction:**

Following the approval of Chimeric Antigen Receptor T-cell Immunotherapy(CAR-T) in multiple countries, the Food and Drug Administration (FDA) approved tumor-infiltrating lymphocytes (TILs) and T-cell receptor-engineered T cells (TCR-T) treatments this year. The utilization of adoptive immunotherapy in tumor treatment has become increasingly prominent. Optimizing the cytotoxic effects of immune cells under *in vitro* culture conditions represents a current hot research topic in this domain.

**Methods:**

In the current experiment, we conducted *in vitro* heat treatment on Jurkat-derived T cells at 39°C. On this basis, we utilized nine distinct injectable solutions and over 70 monomer components of Traditional Chinese Medicine (TCM). Subsequently, we co-cultured these treated Jurkat cells with K562-eGFP cells, and the co-culture process was monitored in real-time using the IncuCyte live-cell analysis system. Equally important, we combined HiMAP high-throughput transcriptome sequencing, proteomics, and metabolomics for in-depth examination. We screened for compounds possessing anti-tumor properties and thoroughly investigated their mechanisms of action.

**Results and Discussion:**

The findings indicated that heating treatment augmented the cytotoxic effect of Jurkat cells against malignant tumors, and the optimal effect was achieved when T cells were exposed to 39°C for a duration of 24 hours(48% increase in cell proliferation rate compared to 37°C treatment). By triggering the generation of heat shock proteins and facilitating mitochondrial energy supply, the 39°C treatment amplified the anti-tumor functions of T cells. By analyzing the data, we identified 3 injectable solutions and more than 20 effective monomers capable of further enhancing the tumor-killing ability of T cells. High-throughput transcriptomics studies disclosed that the combination of thermotherapy and TCM promoted Jurkat cell proliferation, activation, and cytotoxic functions of Jurkat cells, thereby activating the Regulation of mitotic cell cycle to exert anti-tumor effects. The integration of transcriptomic and proteomic data demonstrated that Shengmai Injection significantly enhances the tumor-killing effect of Jurkat cells by down-regulating the Regulation of Apoptosis and Regulation of mitotic cell cycle signaling pathways.

## 1 Introduction

In recent years, tumor immunology has emerged as a groundbreaking advancement and has become a significant breakthrough in cancer treatment, with tumor immunology driving the development of cellular immunotherapy (1, 2). Cellular immunotherapy, also known as adoptive cell therapy (ACT), represents a highly promising strategy in tumor immunology. This approach involves isolating immunologically active cells from tumor patients, expanding and culturing them *in vitro* for functional assessment, and then reinfusing them back into the patient to utilize activated immune cells to kill tumor cells (3, 4). Owing to its advantages, such as precise targeting, significant therapeutic efficacy, minimal side effects, and potential to prevent recurrence and metastasis, ACT has demonstrated unique value in tumor treatment (5). ACT encompasses 3 main forms, all with marketed drugs, including TILs, TCR-T cells, and CAR-T cells (6). Among these, CAR-T therapy was the first to be approved (7). As of March 2024, 10 CAR-T therapies have been approved globally for clinical use in treating hematological malignancies, with 6 targeting the CD19 antigen and 4 targeting the B cell maturation antigen (BCMA). CAR-T cell therapy has shown promising efficacy and prospects in treating hematological malignancies, benefiting tens of thousands of patients. However, there are still many challenges in treating solid tumors (8–11). In a notable development, the FDA approved TCR-T and TIL therapies in January and February 2024, respectively, targeting limited melanoma, marking a significant milestone in applying ACT for solid tumors highlighting its unique therapeutic potential. Nevertheless, ACT faces limitations, including suboptimal efficacy when relying solely on expanded and reinfused autologous cells, difficulties in tumor cell recognition, limited killing capacity, susceptibility to T-cell exhaustion, and poor proliferation within the tumor microenvironment (9, 12, 13). These challenges underscore the urgent need for further research and innovation. Consequently, enhancing the functionality and persistence of T cells has become a focal point of current scientific investigation.

During the COVID-19 pandemic, the impact of fever on the immune system garnered widespread attention. Fever directly affects T cells’ function and promotes immune response by altering the tumor microenvironment. Studies have shown that fever can increase blood flow to tumor tissues, thereby improving drug and immune cell penetration and further enhancing the effectiveness of anti-tumor therapy (14, 15). A study published in the Proceedings of the National Academy of Sciences (PNAS) in 2021 demonstrated that fever enhances the function and efficacy of T cells and that heat-treated T cells were relayed into myeloid leukemia mice, which showed enhanced anti-tumor effects in the mice. The survival of mice was prolonged by injections of 39°C-treated T cells, further suggesting that elevated temperatures can enhance immunity (16). Additionally, a study published in Science Immunology this year pointed out that 39°C can promote the proliferation of T cells. The mechanisms by which fever acts on T cells are comprehensively elucidated (17). This evidence suggests that fever has a potential application in improving the ACT. However, current research primarily focuses on the specific temperature of 39°C and its promotion of T cell proliferation, with no studies yet exploring the effects of temperature gradient changes on T cells.

Modern scientific research has proved that TCM can reconstruct the immunosuppressive cells in the tumor microenvironment and enhance the anti-tumor immune response (18, 19). As early as over 2,000 years ago, TCM theory emphasized the importance of enhancing the body’s immune system function in treating diseases, a process known as strengthening body resistance and consolidating. In clinical applications for cancer treatment, many TCMs exhibit immune-enhancing effects. Many of the monomer components in these TCMs also show potential to enhance the immune system. Studies have shown that monomer components extracted from TCMs can improve anti-tumor immunity and inhibit tumor cell growth and metastasis by reducing the number and function of Treg cells and their secretion of immune-suppressive cytokines (19). For example, ginsenosides, as key components of ginseng, have demonstrated a wide range of beneficial therapeutic effects in clinical applications, and their metabolites have shown anti-tumor activity in *in vitro* experiments (20). Among them, ginsenoside Rg3 and ginsenoside Re are important active ingredients of ginseng, which are widely distributed in the root, stem, and leaf of ginseng. Ginsenoside Rg3 can effectively inhibit the proliferation of cancer cells by inducing apoptosis and down-regulating the EGFR/PI3K/AKT signaling pathway (21). Ginsenoside Re regulates AMPK/TINT in TAMS and inhibits M2 polarization, playing a role in tumor immunotherapy (22). Schisandra chinensis methylin, mainly enriched in Schisandra chinensis fruits, plays a significant role in anti-tumor therapy. Its mechanisms of action include antioxidant, antiapoptotic, anti-inflammatory, and neurotransmitter modulation.4 It can also exert anti-tumor effects by inducing cell cycle arrest at the G_0_/G_1_ phase and inhibiting the expression of the cell cycle protein E (23, 24). However, comprehensive modern research reveals a lack of systematic studies on the combined application of monomer components with ACT.

A moderate 39°C stimulus can enhance T cell function while potentially causing some degree of damage (17). In TCM, the approach to reducing fever not only focuses on lowering body temperature but also emphasizes maintaining and enhancing vital energy. Applying this concept to strategies that activate T cells through heating might allow for maintaining T cell proliferation while further enhancing their function. Temperature-based combination therapy can potentially maintain T cell proliferation while significantly improving their killing capacity, thereby generating a synergistic effect.

In this study, we explored the effects of temperature stimulation and pharmacological agents on the proliferative capacity of Jurkat cells. Building on this study, we investigated whether combining temperature and TCM monomers could synergistically affect Jurkat cell proliferation. This research provides valuable insights into potential strategies to address the challenges of weak proliferative capacity and poor persistence of T cells in ACT. By highlighting the potential of integrating ACT with TCM, this study underscores the importance of combining these approaches as a promising strategy to enhance immune system efficacy. The findings of this study not only contribute to improving the effectiveness of tumor therapy and offer novel directions and ideas for developing personalized treatment regimens. Furthermore, they propose feasible methods to enhance immune cells’ proliferation and cytotoxic capabilities, thereby advancing the field of cellular immunotherapy.

## 2 Materials and methods

### 2.1 Cell culture

Human T-cell Jurkat cells (procured from Beijing Zhongsheng Aobang Biotechnology Co., Ltd.) and human chronic myeloid leukemia cells K562-eGFP (obtained from Hunan Fenghui Biotechnology Co., Ltd.) were cultured in RPMI-1640 complete culture medium supplemented with 10% fetal bovine serum (FBS, Croning) and 1% penicillin/streptomycin (P/S, Gibco). These cells were maintained under humid conditions at 37°C, 38°C, 39°C or 40°C in a 5% CO_2_ atmosphere.

### 2.2 IncuCyte

Dissolve the monomers at the corresponding concentrations in DMSO (**Supplementary Table 1**). Subsequently, the injection solutions and monomer solutions were dissolved at the respective dosages (**Supplementary Table 2**) in the RPMI-1640 complete culture medium to prepare the drug-containing RPMI-1640 complete culture medium. Culture Jurkat cells with the drug-containing medium and incubate the cells in the incubator at the corresponding temperature for 24 hours. After 24 hours, remove the cells and perform complete centrifugation to replace the medium with the normal drug-free 1640 complete culture medium. Seed the cells in a 96-well plate (Costar 3599) (n=6). Place the 96-well plate in the IncuCyte long-term live-cell imaging workstation (IncuCyte@S3) to monitor the proliferation of log-phase Jurkat cells after treatment with different temperature gradients and monomers or injection solutions. Capture an image of each well every two hours at a 4X magnification to observe the dynamic proliferation of cells.

Subsequently, after treating log-phase Jurkat cells with different temperature gradients and monomers or injection solutions for 24 hours, perform complete centrifugation to change the medium, removing the drug-containing medium and replacing it with the normal drug-free RPMI-1640 complete culture medium for co-culture with K562-eGFP cells. To monitor the fluorescence changes, capture four images of each well every two hours at a 4X magnification. Conduct data analysis using the IncuCyte 2020C Rev1 software.

### 2.3 High-throughput 3’ mRNA library construction and sequencing process

From the IncuCyte data, select the injection solutions and monomers that exhibit significant proliferation effects at 39°C. To facilitate a more comprehensive comparison among the monomers, we categorized 35 monomers into seven groups based on their chemical types: glycoside monomer group, flavonoid monomer group, alkaloid monomer group, saponin monomer group, alcohol monomer group, and other monomer groups for transcriptome sequencing. Subsequently, each group of monomers was subjected to the Ingenuity Pathway Analysis (IPA) comparison analysis.

Jurkat cells were seeded at the same concentration in a 96-well plate (FALCON REF353077), treated with different drugs, and incubated at 39°C for 24 hours (n=3). After 24 hours, the cells were removed, centrifuged to discard the drug-containing medium, and resuspended in a 1640 medium. For each sample, 10 µL of the cell suspension was taken and lysed with 10 µL of Cell Lysis Buffer at room temperature for 6 minutes to prepare the cell lysate. For each sample, 2 µL of the cell lysate was taken and mixed with 1 µL of Well Barcode Primer, and the corresponding information between the barcode and the sample was recorded. The mixture was gently vortexed and centrifuged, then placed on a PCR machine for reverse transcription (42°C, 1 hour 30 minutes) to prepare cDNA. For every 24 samples, 9 µL of the reverse transcription sample was taken and placed in a 1.5 mL EP tube, and magnetic beads were added to the mixture for magnetic bead purification. 17 µL of the purified product was mixed with Enzyme E and placed on a PCR machine for enzymatic digestion (37°C, 20 minutes; 80°C, 10 minutes). The digested product was purified again with magnetic beads. Amp Mix1 and Primer Mix1 were added to the purified product for Pre-PCR reaction (98°C, 3 minutes; 98°C, 15 seconds; 65°C, 30 seconds; 72°C, 4 minutes, for four cycles; 72°C, 10 minutes). After magnetic bead purification, 1 µL of the purified product was taken and quantified using a Qubit instrument (Invitrogen Qubit™4 Fluorometer). The nucleic acid concentration should be higher than 1.6 ng/µL.

After nucleic acid quality control, library construction was performed. The purified product was diluted to an appropriate concentration, and 20 ng of the diluted product was taken. Buffer F and Enzyme F were added and placed in a PCR machine for fragmentation and end-repair reaction (37°C, 5 minutes; 65°C, 30 minutes). Adaptor, Mix L1, Mix L2, and adapter dilution solution were added to the reaction product and placed in a PCR machine for adapter ligation reaction (20°C, 15 minutes). The adapter-ligated product was purified for the fourth time with magnetic beads. Amp Mix2 and Primer Mix2/3/4/5 were added to the purified product for PCR amplification reaction (98°C, 30 seconds; 98°C, 10 seconds; 65°C, 1 minute 15 seconds, for 15 cycles; 65°C, 5 minutes), and the corresponding sample information for the Primer was recorded. Nuclease-free water and magnetic beads were added to the amplified product for fragment selection, and purification beads were used to remove fragments of unsuitable sizes, thus obtaining the library.

Using a Qubit instrument, 1 µL of the library was taken, quantified, and checked for fragment size. Libraries that passed quality control were sequenced on the Illumina NovaSeq X-plus sequencing platform in PE150 mode. All HiMAP reagents were purchased from iomics Biosciences Inc.

### 2.4 Untargeted metabolomics

#### 2.4.1 Cell sample processing

Integrating the transcriptomic data and IncuCyte results, select samples with better proliferation effects. A centrifuge was used to collect the suspended cells, and they were quickly washed three times with pre-cooled PBS at 4°C, followed by low-speed centrifugation at 1000g for 1 minute. Discard the supernatant and collect the cell pellet in a 2 mL centrifuge tube. Rapidly freeze the cells in liquid nitrogen for 1-5 minutes and store at -80°C (n=3).

#### 2.4.2 Cell samples will be subjected to analysis on the instrument

Resuspend the samples in prechilled 80% methanol using a vortex mixer. Subsequently, the samples were placed in liquid nitrogen for rapid freezing for 5 minutes. After thawing on ice, vortex for 30 seconds and sonicate for 6 minutes centrifuge at 5000 rpm for 1 minute at 4°C. Collect the supernatant, lyophilize it, and dissolve it in 10% methanol. Take equal volumes from each experimental sample and mix them to create a QC (quality control) sample. A 53% methanol aqueous solution was used as the blank sample. Finally, the solution is injected into the LC-MS/MS system for analysis.

#### 2.4.3 Data processing and metabolite identification

This study processed the raw data files generated by ultra-high-performance liquid chromatography-tandem mass spectrometry (UHPLC-MS/MS) using Compound Discoverer 3.3 (CD3.3, ThermoFisher) software. The processing steps included peak alignment, peak picking, and quantification of each metabolite. The main parameters were set as follows: peak areas were corrected using the first quality control sample (QC1), with an actual mass tolerance of 5 ppm, a signal intensity tolerance of 30%, and a minimum intensity threshold. After initial processing, the peak intensities were normalized to the total spectral intensity.

The normalized data were used to predict molecular formulas based on additive, molecular ion peaks, and fragment ions. Subsequently, the predicted peaks were matched against the mzCloud (https://www.mzcloud.org/), mzVault, and MassList databases to achieve accurate qualitative and relative quantitative results.

For statistical analysis, data processing was conducted using R software (version R-3.4.3), Python (version 2.7.6), and the CentOS operating system (version 6.6). When the data did not conform to a normal distribution, they were standardized using the following formula to obtain relative peak areas:


$$\text{Relative Peak Area = }\frac{\text{Sample Raw Quantitation Value}}{\left(\frac{\sum \text{Sample Metabolite Quantitation Values}}{\sum \text{QC}1\text{ Sample Metabolite Quantitation Values}}\right)}$$

Compounds with a coefficient of variation (CV) of relative peak areas exceeding 30% in the quality control samples were excluded from further analysis. Ultimately, metabolite identification and relative quantification were completed.

#### 2.4.4 Data analysis

These metabolites were annotated using the KEGG database (https://www.genome.jp/kegg/pathway.html), HMDB database (https://hmdb.ca/metabolites) and LIPIDMaps database (http://www.lipidmaps.org/). Principal components analysis (PCA) and Partial least squares discriminant analysis (PLS-DA) were performed at metaX. We applied univariate analysis (t-test) to calculate the statistical significance (P-value). The metabolites with VIP > 1 and P-value< 0.05 and fold change≥2 or FC ≤ 0.5 were considered differential metabolites. Volcano plots were used to filter metabolites of interest based on log2 (Fold Change) and -log10(p-value) of metabolites by ggplot2 in R language.

### 2.5 DIA proteomics

#### 2.5.1 Cell sample processing

Integrating the transcriptomic data and IncuCyte results, select samples with better proliferation effects. Centrifuge at 1000 g for 5 minutes, discard the supernatant, and wash the cell pellet thrice with prechilled PBS solution. The centrifuge is used again to discard the supernatant and collect the cells in a 1.5 mL centrifuge tube. Rapidly freeze the cells in liquid nitrogen and store them in a -80°C freezer (n=3).

#### 2.5.2 Subjecting cell samples to instrumental analysis

Remove the cell samples from the -80°C freezer, add DB protein dissolution solution, and vortex to mix. Sonicate in an ice-water bath for 5 minutes to fully lyse the cells at 4°C, then centrifuge at 12000 g for 15 minutes at 4°C. Collect the supernatant and add an appropriate amount of 1M DTT, followed by incubation in a water bath at 56°C for 1 hour. Cool in an ice bath for 2 minutes, then add sufficient IAM and react in the dark at room temperature for 1 hour.

After protein quantification using the Bradford protein assay kit, take the protein samples and add DB protein dissolution solution (8 M urea, 100 mM TEAB, pH=8.5) to increase the volume to 100 µL. Add trypsin and 100 mM TEAB buffer and mix well. Incubate at 37°C for 4 hours, then add more trypsin and CaCl_2_ and digest overnight. Adjust the pH to less than 3 with formic acid, mix well, and centrifuge at room temperature at 12000 g for 5 minutes. Collect the supernatant and pass it slowly through a C18 desalting column. Wash the column three times with washing solution (0.1% formic acid, 3% acetonitrile), then add an appropriate amount of elution solution (0.1% formic acid, 70% acetonitrile) and collect the filtrate. Lyophilize the filtrate.

Prepare mobile phase A (100% water, 0.1% formic acid) and mobile phase B (80% acetonitrile, 0.1% formic acid). Dissolve the lyophilized powder in 10 µL of mobile phase A, centrifuge at 14000 g for 20 minutes at 4°C, and collect the supernatant. Inject 200 ng of the sample for liquid chromatography-mass spectrometry (LC-MS) analysis.

#### 2.5.3 Data analysis

RAW files were searched and analyzed according to the protein database using the search software DIA-NN. Data analysis was performed in IPA.

### 2.6 IPA analysis

Using R language version 4.4.1 in conjunction with the limma package, datasets underwent standardization, and genes exhibiting |LOG2FC| ≥ 0.25 and P < 0.05 were identified as differentially expressed genes. These genes were subsequently imported into the IPA database provided by QIAGEN Inc. (https://digitalinsights.qiagen.com/IPA). The data were analyzed using QIAGEN IPA, and the results were exported to Cytoscape 3.9.1 for further visualization and network analysis.

The IPA was employed to analyze the canonical pathways of the core regulatory gene set, with IPA assigning Z-scores to each pathway to indicate activation or inhibition status. A Z-score > 0 (orange) signifies an activation state, while a Z-score < 0 (blue) indicates an inhibition state. The study also predicted upstream regulatory factors of the differentially expressed genes, including transcription factors, cytokines, and others. The top 15 activated and inhibited regulatory factors were selected, and their names, molecular types, activation status, Z-scores, P-values, and target gene information were compiled. IPA’s consistency score was calculated to describe the causal consistency between regulatory factors and downstream functional pathways. The network diagram was divided into three parts: differentially expressed genes, regulatory factors, and downstream functional pathways. The IPA-comparison analysis function was used to compare differences between groups, and all networks were ranked by their scores.

### 2.7 Western blotting

Cells were treated in different groups and proteins were extracted. Protein quantification was performed using the BCA Protein Quantification Method (BCA Protein Assay Kit AQ526-500T). After harmonization of protein concentrations, cell lysates were added to 10% TGX Stain-Free Gel (Bio-Rad). Total protein quantification by Image Lab (Bio-Rad) was selected to calculate the normalization factor and analyzed.

## 3 Results

**Figure 1:**
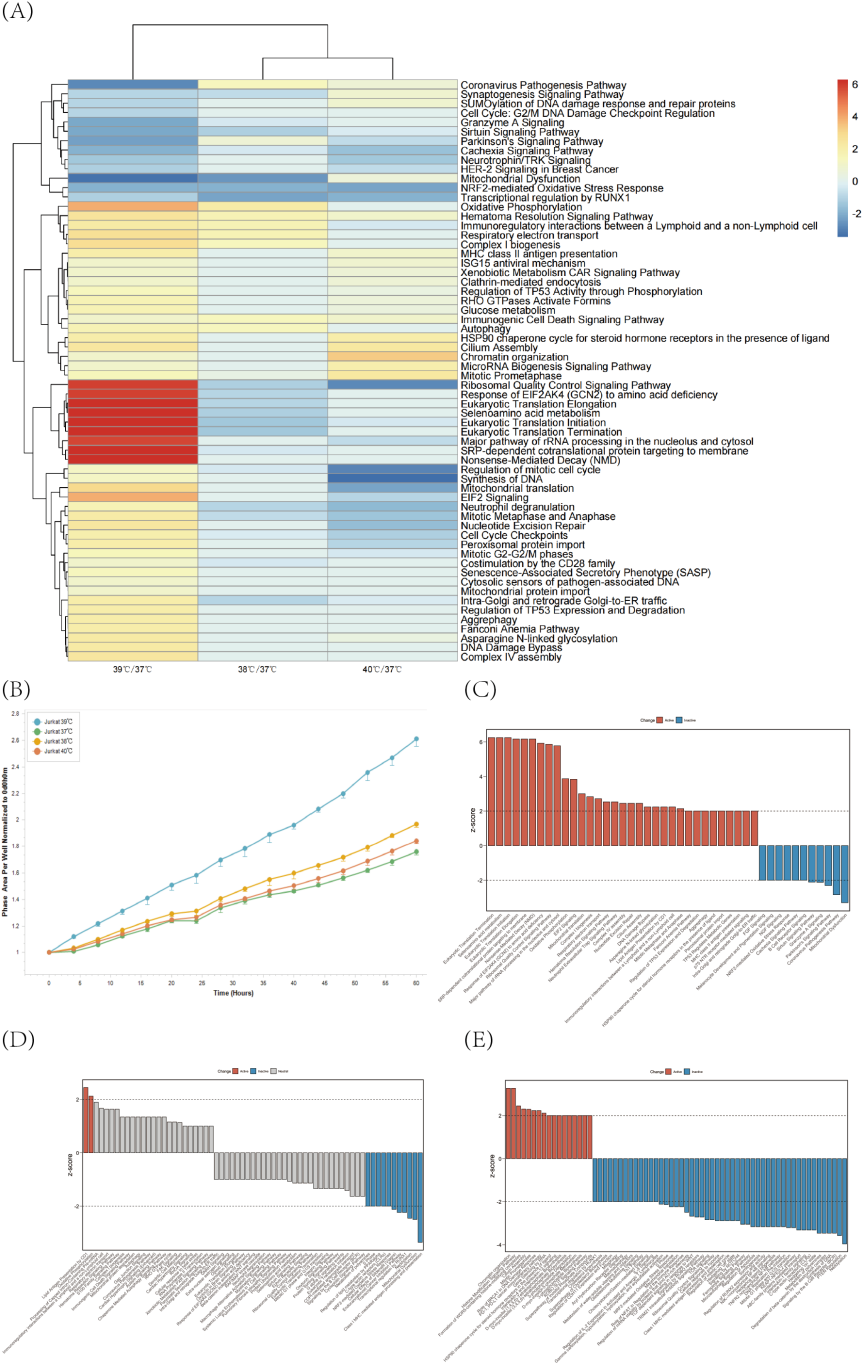
Jurkat cells were cultured at 37°C, 38°C, 39°C and 40°C temperature gradients for 24 h and then assayed to screen the optimal temperature of action for Jurkat cells. **(A)** HiMAP high-throughput transcriptome analysis was performed on Jurkat cells after treatment with different temperatures and intergroup comparisons were made with 37°C conditions. Differences in regulatory pathways at different temperatures relative to 37°C were demonstrated by IPA-comparison analysis function. **(B)** Proliferation profiles of Jurkat cells after treatment with different temperature gradients were monitored using the IncuCyte Long-Term Live Cell Workstation. **(C–E)** Graphs showing the results of major regulatory pathway activities at 39°C, 38°C, and 40°C compared to 37°C.

Jurkat cells in the logarithmic growth phase were selected, cultured in T25 Cell Culture Flasks and placed in a 5% CO_2_ incubator with different temperature gradients at 37°C, 38°C, 39°C and 40°C for 24 hours. The treated cells were placed in the IncuCyte Real-Time Live Cell Analysis System to detect changes in the proliferative capacity of Jurkat cells after treatment with different temperatures. Compared with 37°C, the proliferation of Jurkat cells was promoted to some extent after temperature treatments at 38°C, 39°C, and 40°C, with the temperature intervention at 39°C having the most significant effect on the promotion of Jurkat cell proliferation (**Figure 1B**). In order to investigate the mechanism of action and the effects of different temperature treatments on Jurkat cells deeply, we performed HiMAP high-throughput transcriptome banking and sequencing of Jurkat cells after four different temperature treatments, respectively. In the analysis, we first compared all other groups with the 37°C group to identify the differences from 37°C after different temperature treatments. Subsequently, sorting based on z-score scores by the comparison function of IPA showed significant differences in the enhancement and inhibition of pathway activity by these temperatures. The major pathways upregulated in Jurkat cells after 39°C treatment include immunoregulatory interactions between lymphocytes and non-lymphocytes, ribosomal quality control signaling pathways, and MHC class II antigen presentation, which promote DNA replication and recombination and repair, cellular assembly and organization, and cellular function and maintenance, which are important in maintaining the normal physiological functions of Jurkat cells and in response to various internal and external environmental changes. In contrast, the major pathways down-regulated in Jurkat cells after treatment at 39°C include Mitochondrial Dysfunction and DNA damage, which alter enzyme activity and protein stability, thereby affecting the function and maintenance of Jurkat cells (**Figure 1A**). We continued the cluster analysis of each temperature-treated group by IPA software with z-score value as the filtering condition, and the results showed that the activity of signaling pathways such as Eukaryotic Translation Termination, Selenoamino acid metabolism and Eukaryotic Translation Elongation was significantly enhanced, while the activity of signaling pathways such as Mitochondrial Dysfunction and Sirtuin Signaling Pathway was suppressed after the temperature treatment of 39°C. By regulating these pathways, the processes such as translation termination in Jurkat cells were affected, which in turn promoted the growth of Jurkat cell numbers (**Figure 1C**). After 38°C treatment, signaling pathways such as Class I MHC mediated antigen processing and presentation and Transcriptional regulation by RUNX1 were down-regulated, which could potentially affect the function of Jurkat cells (**Figure 1D**). After 40°C treatment, signaling pathways such as Role of BRCA1 in DNA Damage Response, Histone Modification Signaling Pathway and Formation of WDR5-containing histone-modifying complexes were enhanced in their activity, whereas signaling pathways such as Neddylation and Synthesis of DNA were suppressed in their activity, and the alteration of the activity of these signaling pathways might also affect the function of Jurkat cells (**Figure 1E**).

**Figure 2:**
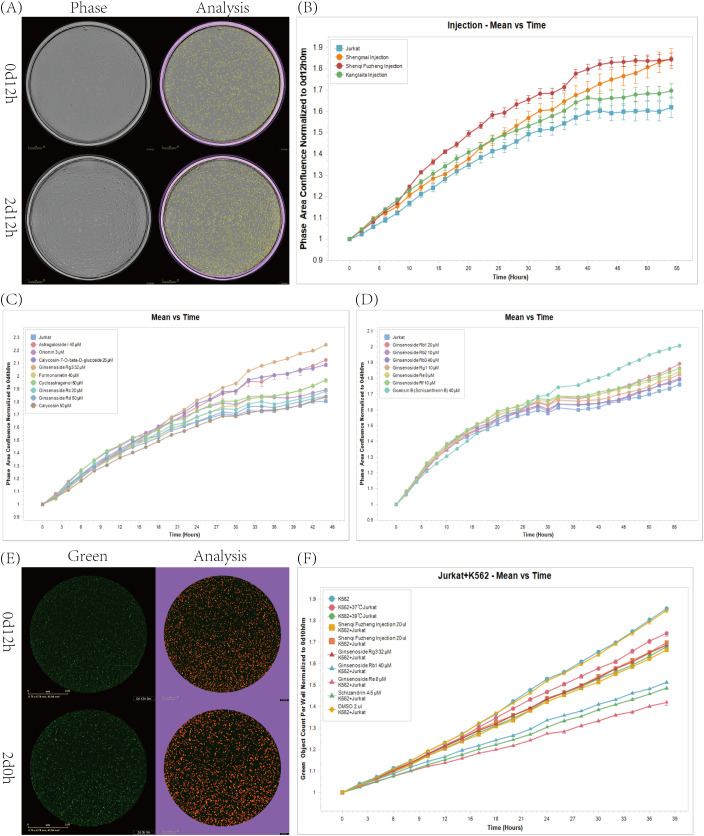
Effects of injections, monomers, and their combination with temperature on Jurkat cell proliferation. **(A)** IncuCyte detects cell proliferation in real-time Phase images as well as cell recognition images. **(B)** Impact of some approved injections on Jurkat cell proliferation at 39°C. (n=6). **(C, D)** Impact of some monomers on Jurkat cell proliferation at 39°C. (n=6). **(E)** Real-time Green images of IncuCyte detection of cell killing and images of cell recognition. **(F)** Comparison of the killing capabilities of T cells and tumor cells after treatment with drugs that promote Jurkat cell proliferation. (n=6).

We selected TCM injections approved by the Center For Drug Evaluation and widely used in clinical oncology. Using the IncuCyte live-cell analysis system, we assessed their effects on T cell proliferation. (n=6) The results showed that injections such as Shengmai Injection, Shenqi Fuzheng Injection, and Kanglaite Injection significantly promoted Jurkat cell proliferation (**Figures 2A, B**; **Supplementary Video 1**). To further explore the main active components, we reviewed relevant literature and selected several monomers (Complete monomer details are provided in **Supplementary Table 1**) to study their effects on Jurkat cell proliferation. We found that Ginsenoside Rb2, Gomisin B (Schisantherin B), Astragaloside, Calycosin-7-0-beta-D-glucoside, GinsenosideRg3 monomers had a significant promoting effect on Jurkat cell proliferation (**Figures 2C, D**). To further demonstrate that the combination of TCM and 39°C treatment not only promotes proliferation but also enhances Jurkat cell killing of tumors, we co-cultured K562-eGFP cells transfected with GFP with Jurkat cells and measured the area of GFP-positive cells to assess Jurkat cell killing of K562-eGFP. The results showed that Ginsenoside Rg3, Ginsenoside Rb1, Ginsenoside Re, Schizandrin A monomers had a promoting effect on Jurkat cells (**Figures 2E, F**; **Supplementary Figure 1**).

**Figure 3:**
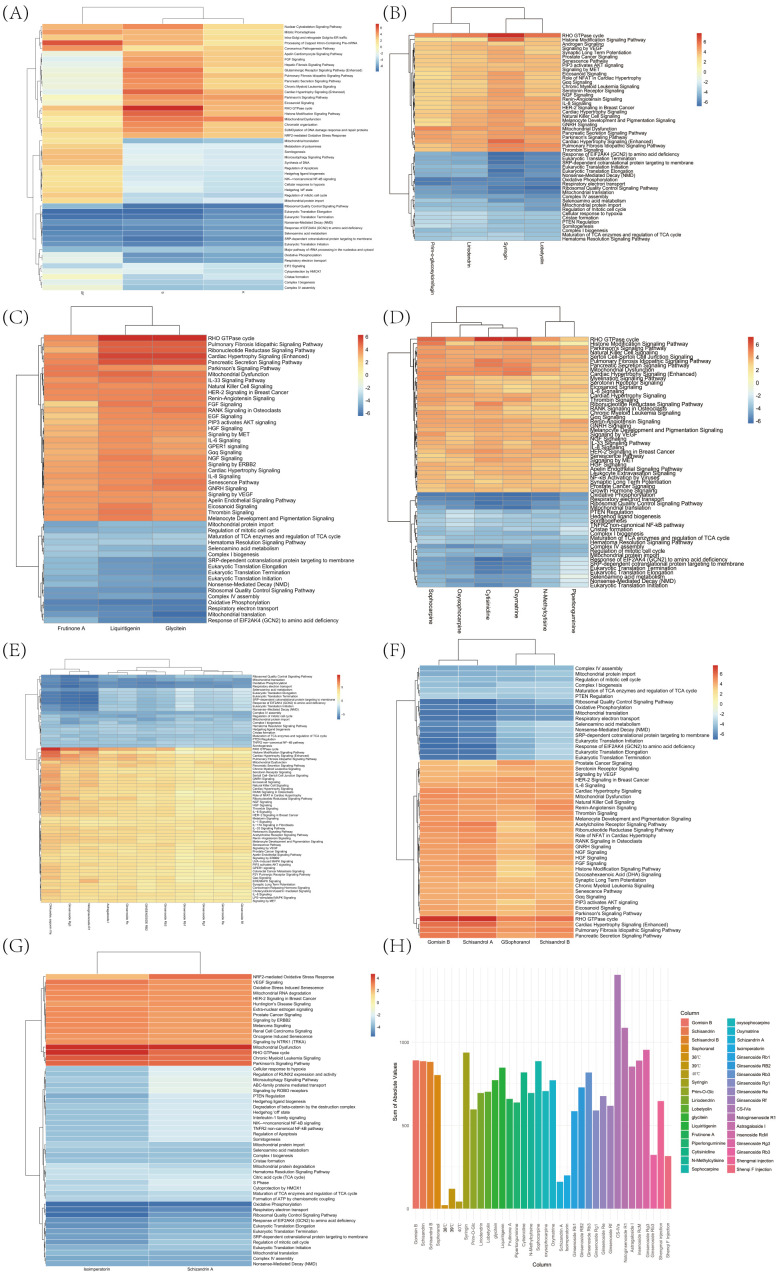
HiMAP sequencing results and scores. **(A)** Based on the IPA comparison function, a comparison was made between the effects of 39°C combined injection fluid and 39°C treatment on Jurkat cells. **(B)** Utilizing the IPA comparison function to assess the comparative effects of 39°C combined glycoside monomers and DMSO treatment on Jurkat cells. **(C)** Employing the IPA comparison functionality to assess the comparative effects of 39°C combined flavonoid monomers versus DMSO treatment on Jurkat cells. **(D)** Utilizing the IPA comparison functionality to contrast the effects of 39°C combined alkaloid monomers with DMSO treatment on Jurkat cells. **(E)** Employing IPA comparison functionality to assess the comparative effects of 39°C combined saponin monomers versus DMSO treatment on Jurkat cells. **(F)** Utilizing the IPA comparison functionality to evaluate the comparative effects of 39°C combined alcohol monomers versus DMSO treatment on Jurkat cells. **(G)** Employing the IPA comparison functionality to evaluate the comparative effects of 39°C combined alkaloid monomers versus DMSO treatment on Jurkat cells. **(H)** The effects of various traditional Chinese medicine injections and monomers on Jurkat cells are illustrated through the assessment of IPA z-scores.

In the aforementioned experiments, we have screened from over 10 TCM injections and more than 80 monomers of TCM, identifying 3 injections and over 20 monomers that promote the proliferation of Jurkat cells. To elucidate the specific mechanisms by which these TCM injections and monomers enhance Jurkat cell proliferation at 39°C, we conducted high-throughput transcriptome sequencing on these drug-treated Jurkat cells using HiMAP technology (n=3). Transcriptome heatmap results indicate that the three types of injections uniformly upregulate the Mitotic Prometaphase, FGF Signaling, and Eicosanoid Signaling pathways, which are known to promote the proliferation and activation of Jurkat cells, as well as the secretion of inflammatory factors, potentially representing a common mechanism of action for immune-modulating injections. Concurrently, we observed that the Shengmai Injection can downregulate the Regulation of Apoptosis, thereby inhibiting programmed cell death in Jurkat cells, preventing cell exhaustion, and promoting proliferation, significantly enhancing the cytotoxic effects of Jurkat cells on tumors. The transcriptome heatmap results demonstrate that the three types of injections uniformly upregulate the Eicosanoid Signaling pathway, thereby enhancing the inflammatory response of Jurkat cells and significantly improving their cytotoxic effects on tumors (**Figure 3A**). Subsequently, each group of monomers was subjected to IPA-comparison analysis (**Figures 3B–G**). To further characterize the systemic effects of various traditional Chinese medicines on Jurkat cells, we have developed a scoring system based on the IPA z-score to illustrate the comprehensive impact of each medication on Jurkat cells (**Figure 3H**). Through IPA pathway enrichment and clustering analysis, we found that the saponin monomer group generally upregulates the IL-8 Signaling, Eicosanoid Signaling, IL-33 Signaling Pathway, IL-17A Signaling in Fibroblasts, IL-1 Signaling pathways, and NGF Signaling, ERK/MAPK Signaling, LPS-stimulated MAPK Signaling, PIP3 activates AKT signaling, Signaling by ERBB2 pathways, while downregulating the PTEN Regulation pathway, thereby promoting the proliferation and activation of Jurkat cells, enhancing their recognition and cytotoxic capabilities against tumors (**Figure 3E**). Glycoside monomer group, flavonoid monomer group, alkaloid monomer group, and alcohol monomer group uniformly upregulate the Eicosanoid Signaling and PIP3 activates AKT signaling pathways while downregulating the Regulation of mitotic cell cycle pathway, thereby promoting the activation of Jurkat cells, the secretion of inflammatory factors, and proliferation. This subsequently enhances the cytotoxic capabilities of Jurkat cells against tumors (**Figures 3B–D, F**). In the other monomer group, Schisandrin A significantly upregulates the Signaling by ERBB2 pathway and downregulates the Regulation of Apoptosis pathway, promoting the proliferation of Jurkat cells, inhibiting apoptosis and exhaustion, and improving the cytotoxic effects of Jurkat cells on tumor cells (**Figure 3G**).

**Figure 4:**
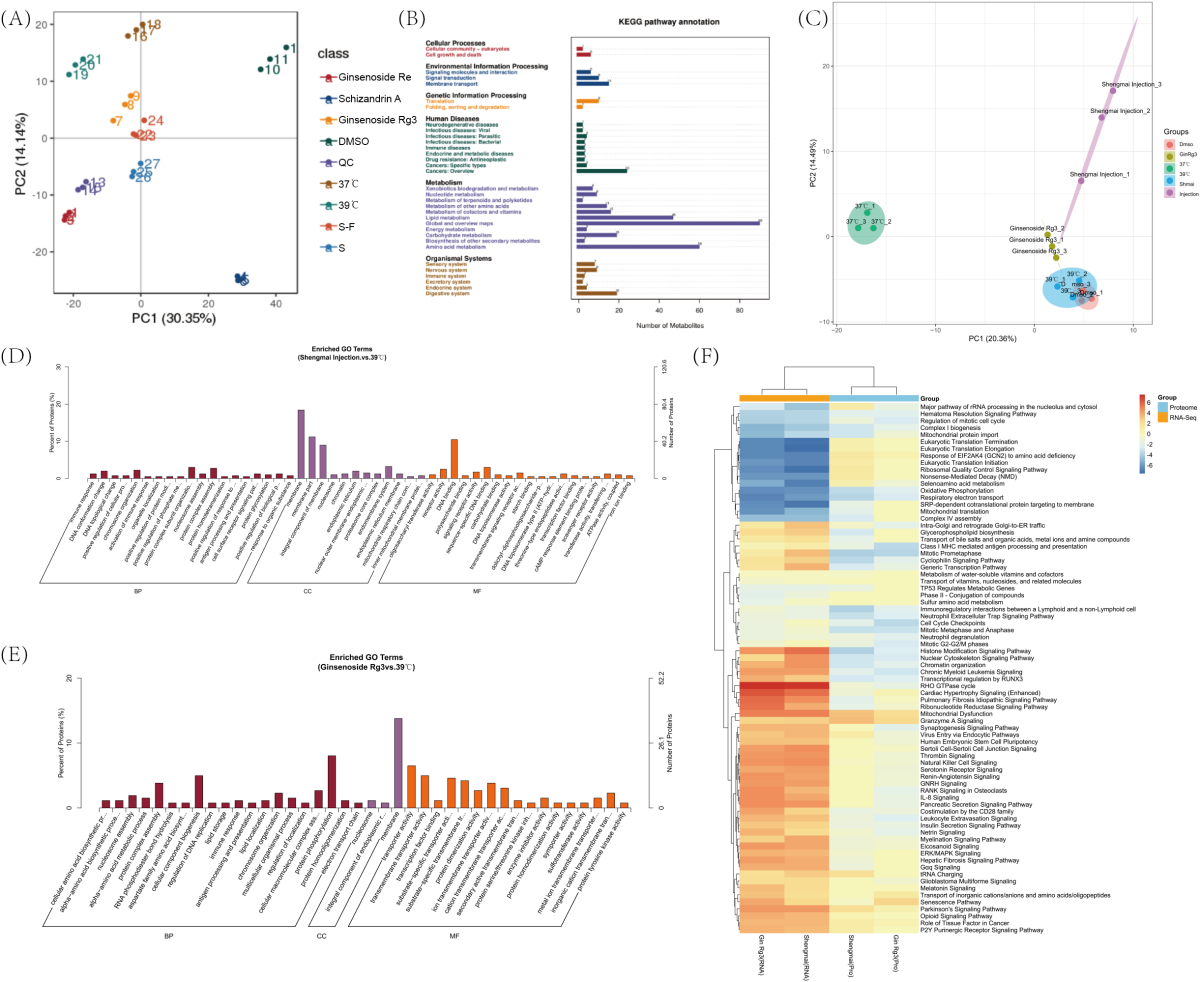
Metabolomics and proteomics results. **(A)** Metabolomic principal component analysis (PCA) clustering of Jurkat cells across various experimental groups. **(B)** Metabolomic KEGG pathway enrichment results for Jurkat cells across various experimental groups. **(C)** Proteomic principal component analysis (PCA) clustering of Jurkat cells across various experimental groups. **(D)** Proteomic GO enrichment analysis of Jurkat cells treated with ginsenoside Rg3 in combination with DMSO and separately with a 39°C Celsius treatment. **(E)** Proteomic Gene Ontology (GO) enrichment analysis of Jurkat cells treated with Shengmai injection in combination with DMSO and separately with a 39°C Celsius treatment. **(F)** Utilizing the IPA comparison functionality, a correlative analysis of the transcriptome, and proteome was conducted in Jurkat cells treated with temperature in conjunction with ginsenoside Rg3, Shengmai injection, and DMSO, respectively.

To further investigate the effects of the more effective injections and monomers on Jurkat cells, we selected ginsenoside Rg3, ginsenoside Re, schizandrin A monomer, Shengmai Injection, and Shenqi Fuzheng Injection for metabolomic analysis, and performed proteomic analysis on ginsenoside Rg3 monomer and Shengmai Injection. PCA clustering analysis revealed that the metabolomic results had good inter-group discrimination (**Figure 4A**). Each monomer, when combined with a 39°C treatment, significantly impacts the metabolic pathways of Jurkat cells, primarily focusing on pathways involved in Cell growth and death, Lipid metabolism, Amino acid metabolism, and Cancers. These alterations in metabolism demonstrate the changes in Jurkat cell metabolism following treatment with the selected drugs, which are conducive to promoting Jurkat cell proliferation, inhibiting apoptosis and exhaustion, and enhancing the cytotoxic effects of Jurkat cells against tumor cells (**Figure 4B**). PCA of the proteome revealed a high degree of intra-group similarity and distinct inter-group discrimination (**Figure 4C**). The GO enrichment analysis of the proteome indicated that ginsenoside Rg3 modulates the biological process of immune response and the molecular function of transmembrane transporter activity, thereby enhancing the immunological response and the secretion of inflammatory cytokines in Jurkat cells (**Figure 4D**). The proteomic GO enrichment results of Shengmai Injection revealed the presence of ‘immune response’ and ‘positive regulation of cellular protein’ biological processes, which contribute to the activation of Jurkat cells and the secretion of inflammatory factors (**Figure 4E**). These findings exhibit a high degree of similarity with the outcomes of our drug screening, further substantiating the effects of the monomer and the injectable solution. Utilizing the IPA-comparison tool, we conducted a correlative analysis of the transcriptome and proteome for ginsenoside Rg3 monomer and Shengmai Injection. The results indicated that both omics approaches exerted an impact on the ‘Regulation of mitotic cell cycle’ and ‘Neutrophil Extracellular Trap Signaling Pathway’ pathways, which are conducive to the proliferation of Jurkat cells, enhancement of their inflammatory response, and subsequently, the augmentation of their cytotoxic effects on tumor cells (**Figure 4F**).

**Figure 5:**
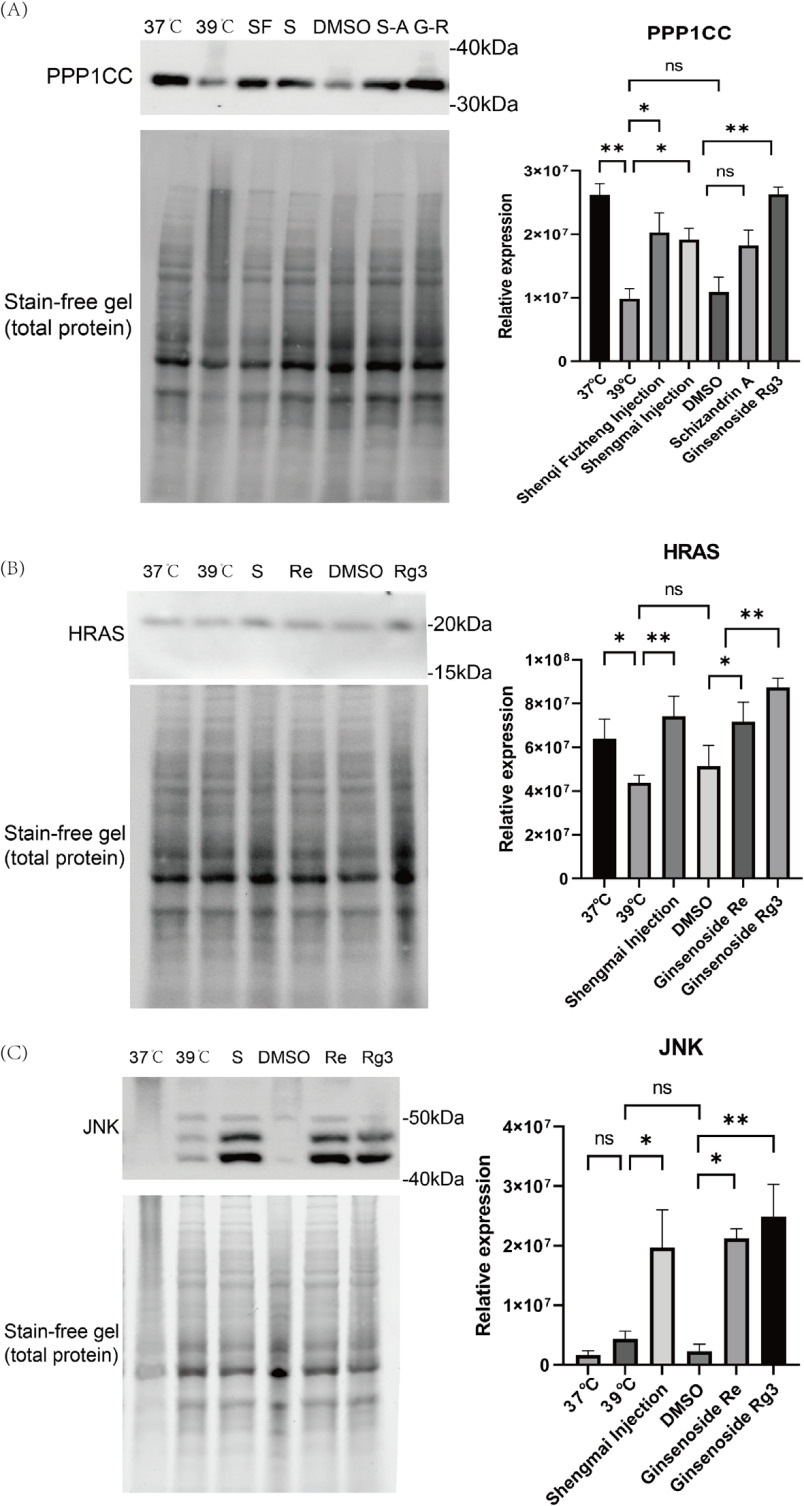
Western blotting results. **(A)** The expression of PPP1CC protein in Jurkat cells treated with different conditions was detected by Western blot. Protein expression was quantified by Image Lab software. (n=3). **(B)** The expression of HRAS protein in Jurkat cells treated with different conditions was detected by Western blot. Protein expression was quantified by Image Lab software. (n=3). **(C)** The expression of JNK protein in Jurkat cells treated with different conditions was detected by Western blot. Protein expression was quantified by Image Lab software. (n=3). *p < 0.05, **p < 0.01.

Based on the cell proliferation results, as well as the HIMAP transcriptome, proteome, and metabolome results, we selected two pathways, mitosis and FGF signaling, and picked the corresponding proteins for validation (n=3). PPP1CC is a protein phosphatase that plays an important role in the cell cycle and proliferation, especially in the regulation of mitosis.39°C treatment of cells for 24 h resulted in a decrease in the expression of PPP1CC relative to that of 37°C (p<0.01). 39°C treatment of cells with a combination of Shenqi Fuzheng Injection and Shengmai Injection the expression of PPP1CC was enhanced relative to 39°C treatment (p < 0.05). Since the monomers were dissolved in DMSO reagent, we compared cells treated with different monomers to the DMSO group. The results showed that there was no significant difference in Schizandrin A combined with 39°C relative to DMSO, and PPP1CC expression was significantly higher in Ginsenoside Rg3 compared to DMSO treatment (P < 0.01). (**Figure 5A**). HRAS is a member of the small GTPase family, and HRAS regulates cell proliferation, differentiation and survival by modulating downstream signaling pathways. The expression of HRAS was slightly decreased in 39°C treated cells for 24h relative to 37 degrees (P<0.05). Shengmai Injection combined with 39°C treatment significantly increased the expression of HRAS compared to 39°C treatment (P<0.01). DMSO was not significantly different relative to 39°C. Ginsenoside Re and Ginsenoside Rg3 combined with 39°C showed significant elevation of HARS relative to DMSO (P<0.05,P<0.01). (**Figure 5B**). JNK belongs to the stress-activated mitogen-activated protein kinase (MAPK) family. FGF can indirectly activate the JNK pathway through the activation of FGFR.JNK affects the expression of cell cycle genes through the regulation of the activity of transcription factors, which in turn regulates cell proliferation. There was no significant difference in JNK expression in 39°C-treated cells versus 37°C, and no significant difference in DMSO combined with 39°C versus 39°C. Shengmai Injection combined with 39°C elevated the expression of JNK compared to 39°C (P < 0.05). Ginsenoside Re and Ginsenoside Rg3 combined temperature treatments showed an increase in the expression of JNK compared to DMSO (P<0.05,P<0.01). (**Figure 5C**).

## 4 Discussion

The Jurkat cell line, one of the most commonly used T cell lines, is characterized by their ease of cultivation, rapid proliferation, and the ability to mimic various functions of T cells, making them widely employed in studies simulating T cell functions and related research (22, 23). In the preliminary stages of drug screening, Jurkat cells can serve as a substitute for T cells derived from peripheral blood mononuclear cells (PBMCs) to some extent (24). However, despite their many similarities to T cells, Jurkat cells exhibit relatively weak cytotoxic functions. This limitation is particularly evident when assessing the impact of drugs on T cell cytotoxic activity. In subsequent studies, drugs identified through screening can be further validated for their cytotoxic activity using PBMC T cells. This approach allows for a more accurate evaluation of the actual effects of drugs on T cell cytotoxic activity, thereby ensuring the reliability of the screening results. Moreover, since the Jurkat cell line is of tumor origin, drugs that inhibit its proliferation may concurrently exhibit certain anticancer activities. This finding opens new avenues for further exploration in subsequent research.

This study systematically analyzes the effects of different temperatures on T cell proliferation and transcriptomic levels, initially revealing the underlying mechanisms, which provide insights and guidance for subsequent research. The results indicate that 39°C has the most significant positive impact on T cells. Through western blot experiments, we observed the effects of different temperatures, injection solutions and monomers on Jurkat cell production. The results of the experiment showed that while the 39 degree temperature treatment promoted cell proliferation, it also had some effects on the cells. While the drug combined with temperature treatment showed a significant increase in protein expression. The biological mechanisms behind this phenomenon remain unclear and warrant further in-depth investigation, offering new perspectives and approaches for the treatment of T cell-related diseases.

This study innovatively combines TCM injections and TCM monomers with temperature. The study screened herbal injections and monomers that can promote the proliferative capacity of T cells through proliferation assays and demonstrated that these drugs can further enhance T cell function after treatment at 39°C. The screened drugs can subsequently be combined with other methods to further enhance T cell function, such as PD-1 monotherapy or combined with immune factor therapy, which provides a paradigm for the research of various immunotherapies assisted by TCMs.

The injection solutions selected in this study are all commonly used in clinical cancer treatment. These injection solutions can directly enter the body, facilitating cellular uptake and providing convenient conditions for our *in vitro* experiments. However, the traditional oral administration of TCM has certain limitations in *in vitro* research. Its components need to go through the complex metabolic process of the digestive system before entering the bloodstream. This makes it difficult to simulate their direct effects on cells *in vitro*, and it is challenging to precisely control the drug concentration and duration of action, thereby affecting the in-depth exploration of the drug’s mechanism of action. In the future, the administration methods of TCM can be further improved to better integrate TCM with cancer treatment.

HiMAP high-throughput transcriptome technology can detect a large number of transcripts in parallel within a short time and rapidly obtain the sequence and expression information of almost all the transcripts of the cells or tissues in specific states, making the transcriptome information available to the researchers and the researchers. Information and expression information, allowing transcriptome sequencing to be performed with less than 10,000 immune cells (i.e., the number of cells in 1 well of a 96-well plate) and greatly reducing experimental costs. In addition, the ability of IPA-comparison to compare changes in the activity of different pathways, coupled with the high accuracy of the results provided by its database, opens up a new pathway for transcriptome drug screening, making it practically feasible and operational and greatly facilitating the application and development of transcriptome technology in the field of drug screening. In addition, the combination of IPA-comparison function makes transcriptome drug screening possible. Our pioneering scoring system also demonstrates a good fit with drug efficacy and has a wide range of application prospects.

In summary, this study utilized the IncuCyte Long-Term Live-Cell Work station and HiMAP high-throughput transcriptome sequencing technology to successfully identify drugs that further promote T cell proliferation under 39°C conditions and elucidated the mechanisms by which these drugs, in combination with temperature, enhance T cell proliferation. This research not only provides innovative approaches and methods for the study of TCM in adjuvant immunotherapy but also demonstrates the immense potential of high-throughput transcriptome sequencing technology in drug screening and efficacy elucidation, opening new avenues for efficient and low-cost drug screening methods. Additionally, this study lays a solid foundation for subsequent in-depth analysis of the specific pharmacological mechanisms and immunotherapy targets of TCM in disease prevention and treatment, providing valuable theoretical support and practical guidance for the further development of tumor immunotherapy.

## Data Availability

The data presented in the study are deposited in the NGDC repository, accession number PRJCA037024.
