# Supplementary material for: Screening and mechanistic study of natural compounds that enhance T cell anti-tumor effects post-heat treatment
Source: Front Immunol. 2025 Mar 27;16:1537398. doi: 10.3389/fimmu.2025.1537398 (PMC11983556; doi:10.3389/fimmu.2025.1537398)
Supplement: Supplementary file 1 [file DataSheet1.docx]

Supplementary Material

# Supplementary Tables


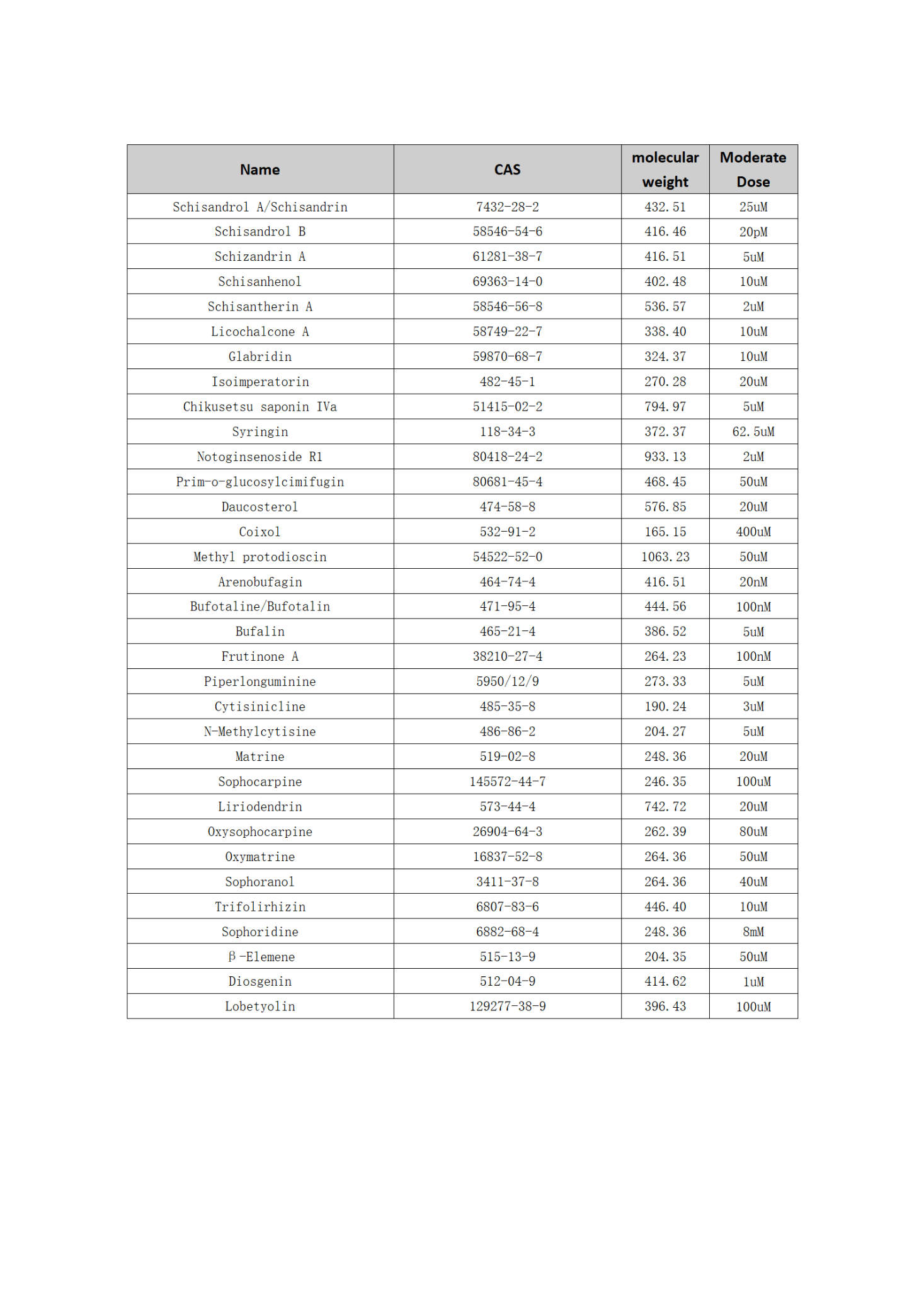


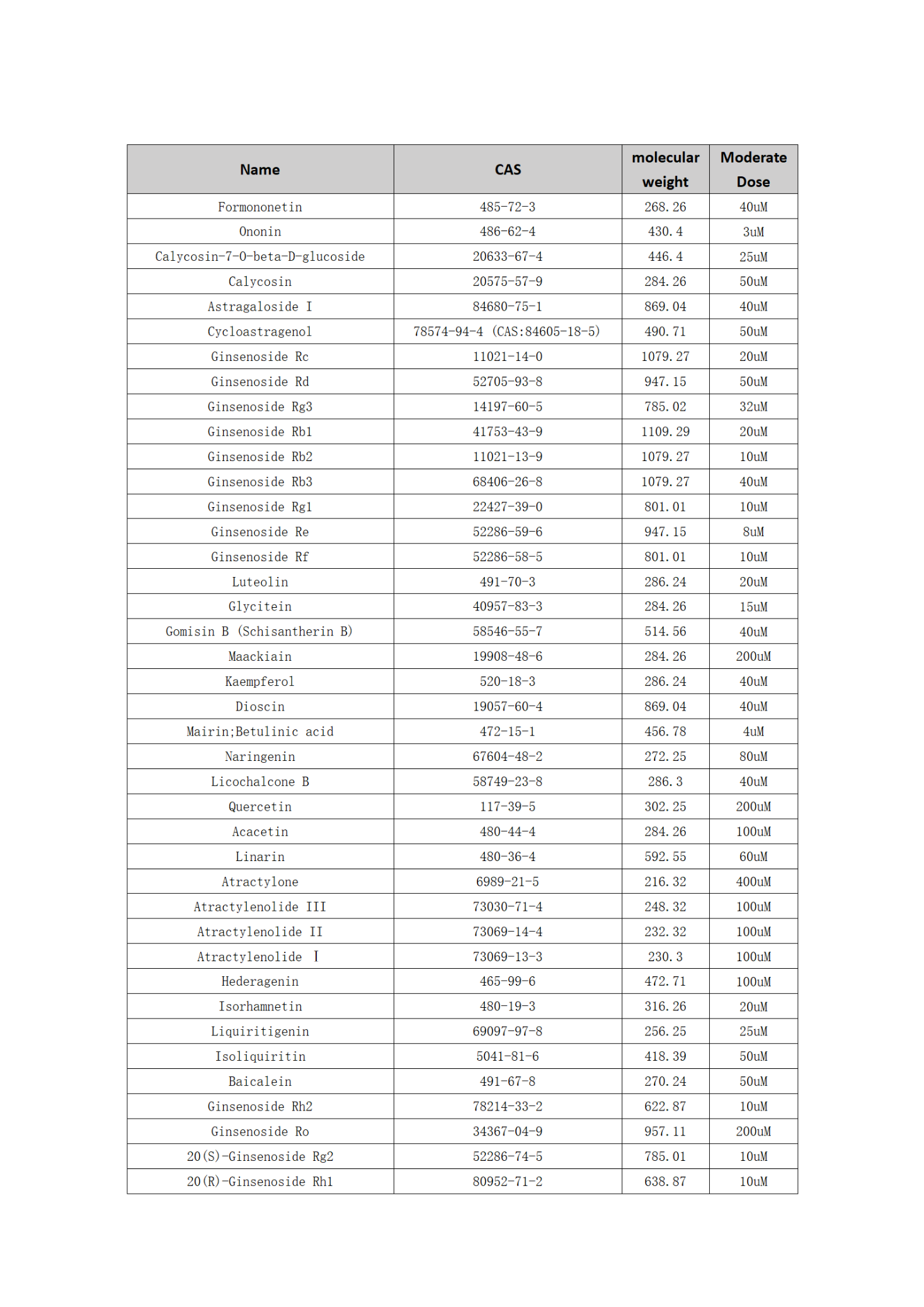


**Table S1.** Names and dosages of all monomers used in the experiment.


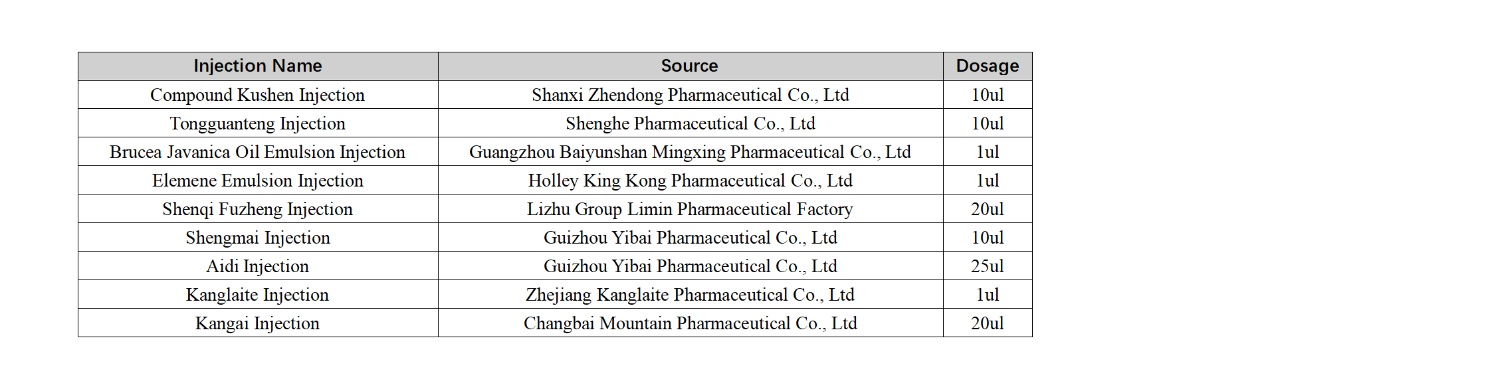


**Table S2.** Names and dosages of all injections used in the experiment.

# Supplementary Figures


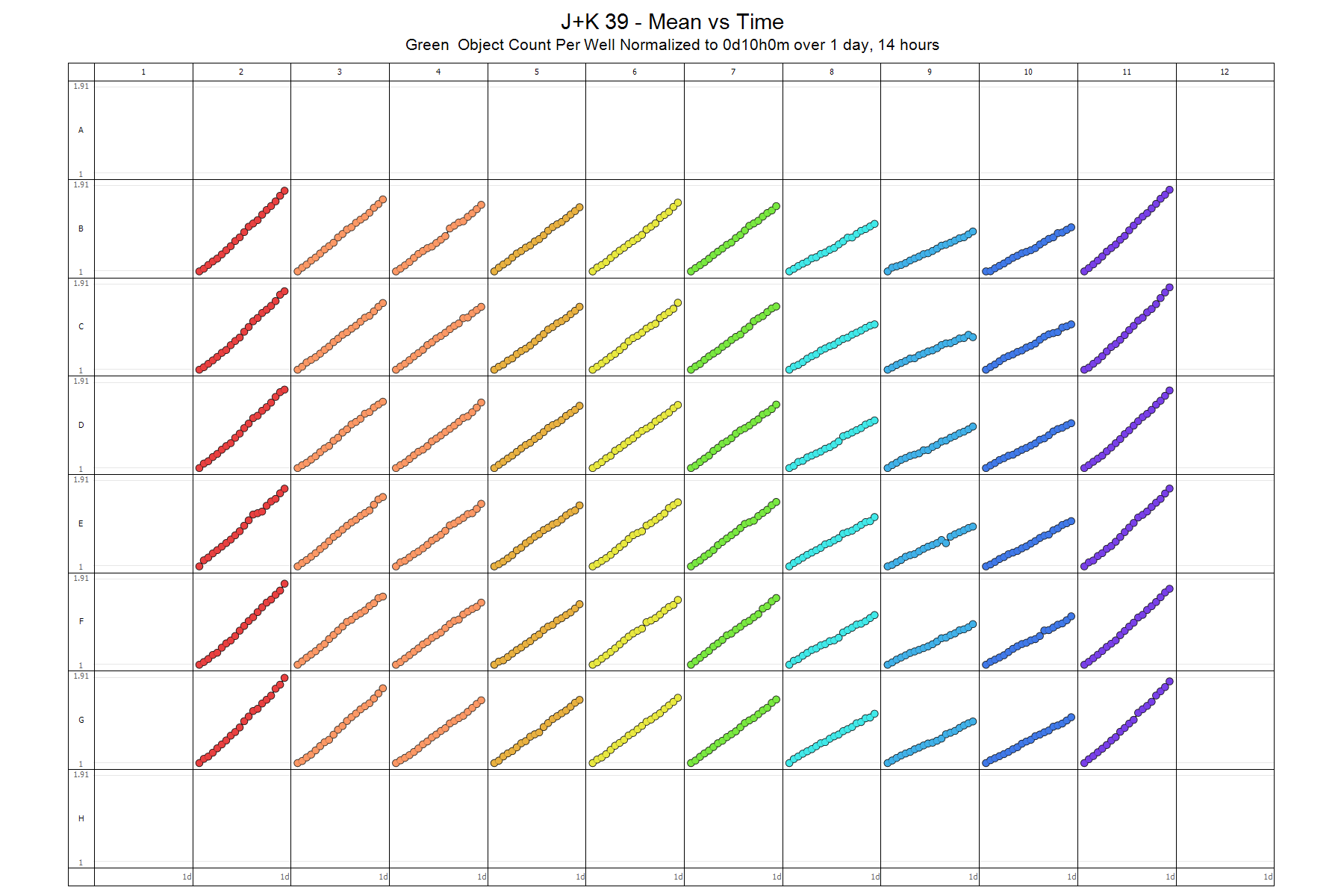


**Figure S1.** Growth curves for each cell at each time point in the IncuCyte system (n=6).

# Supplementary Videos

**Video S1.** Cell growth in the IncuCyte system.
